# Evaluation of fully automated cephalometric measurements obtained from web-based artificial intelligence driven platform

**DOI:** 10.1186/s12903-022-02170-w

**Published:** 2022-04-19

**Authors:** Ravi Kumar Mahto, Dashrath Kafle, Abhishek Giri, Sanjeev Luintel, Arjun Karki

**Affiliations:** Department of Orthodontics and Dentofacial Orthopedics, Kathmandu University School of Medical Sciences, Dhulikhel, Nepal

**Keywords:** Artificial intelligence, Lateral cephalogram, WebCeph™

## Abstract

**Background:**

Artificial Intelligence has created a huge impact in different areas of dentistry. Automated cephalometric analysis is one of the major applications of artificial intelligence in the field of orthodontics. Various automated cephalometric software have been developed which utilizes artificial intelligence and claim to be reliable. The purpose of this study was to compare the linear and angular cephalometric measurements obtained from web-based fully automated Artificial Intelligence (AI) driven platform “WebCeph”™ with that from manual tracing and evaluate the validity and reliability of automated cephalometric measurements obtained from “WebCeph”™.

**Methods:**

Thirty pre-treatment lateral cephalograms of patients were randomly selected. For manual tracing, digital images of same cephalograms were printed using compatible X-ray printer. After calibration, a total of 18 landmarks was plotted and 12 measurements (8 angular and 4 linear) were obtained using standard protocols. The digital images of each cephalogram were uploaded to “WebCeph”™ server. After image calibration, the automated cephalometric measurements obtained through AI digitization were downloaded for each image. Intraclass correlation coefficient (ICC) was used to determine agreement between the measurements obtained from two methods. ICC value < 0.75 was considered as poor to moderate agreement while an ICC value between 0.75 and 0.90 was considered as good agreement. Agreement was rated as excellent when ICC value > 0.90 was obtained.

**Results:**

All the measurements had ICC value above 0.75. A higher ICC value > 0.9 was obtained for seven parameters i.e. ANB, FMA, IMPA/L1 to MP (°), LL to E-line, L1 to NB (mm), L1 to NB (°), S-N to Go-Gn whereas five parameters i.e. UL to E-line, U1 to NA (mm), SNA, SNB, U1 to NA (°) showed ICC value between 0.75 and 0.90.

**Conclusion:**

A good agreement was found between the cephalometric measurements obtained from “WebCeph”™ and manual tracing.

## Introduction

Cephalometric analysis is an indispensable tool for orthodontics diagnosis, treatment planning and evaluating treatment results. Manual cephalometric tracing and analysis despite being “gold standard” is cumbersome, time consuming and can be associated with measurements and calculation errors in addition to errors occurring due to human fatigue. To overcome these problems, computerized cephalometric analysis software were developed. Most of these softwares can do multiple cephalometric analyses as accurately as human experts in a very short period of time once the landmarks are identified and plotted manually on digital images of cephalogram [1–9].

Artificial Intelligence (AI) is the science and engineering of making intelligent machines, especially intelligent computer programs [10]. In recent years, software employs AI for automatic landmark identification instead of manual identification. These fully automatic softwares can dramatically reduce the time and effort of orthodontists involved in the orthodontic case analysis and diagnosis [11]. Since landmark identification is one of the major sources of error in cephalometric analysis, it is essential to evaluate the accuracy of cephalometric measurements obtained through automatic landmark detection feature of commercially available fully automated cephalometric analysis software like CephX®, CEFBOT, WebCeph™, etc. Recently, few authors have evaluated the accuracy of web-based fully automated cephalometric analysis software CephX® and CEFBOT [12–14]. However, we could not find any published study evaluating the cephalometric measurements obtained from “WebCeph”™. “WebCeph”™ is a web-based fully automated AI driven platform that can perform nine different cephalometric analysis and two composite analysis along with interpretation based on obtained cephalometric measurements. In addition, it can be used to store and maintain archive of digital images of patient’s cephalogram, orthopantomogram and photographs. Additionally, it has features like visual treatment simulation and superimposition which are really useful during day-to-day orthodontic practice. The present study aimed to compare the linear and angular cephalometric measurements obtained from WebCeph™ with manual cephalometric analysis and evaluate the accuracy of automated cephalometric measurements obtained from WebCeph™.

## Methods

This study was approved by the Institutional Review Committee, Kathmandu University School of Medical Sciences (IRC no: - 48/2021) and was conducted in accordance with the principles of the Declaration of Helsinki. Informed consent was obtained from all the subjects and/or their legal guardians(s). Calculation of sample size was done based on previous studies by Alqahtani [12] and Silva et al. [14]. A total of 30 pretreatment lateral cephalograms of patients (8 males, 22 females, mean age: 20.17 ± 6.72 years) meeting the inclusion criteria were randomly selected from the archive of Department of Orthodontics, Dhulikhel Hospital. No differentiation was made for chronological/skeletal age, gender type of malocclusion and/or skeletal pattern.

### Inclusion criteria

1. Good quality cephalograms of patients without any artifacts that might interfere with the location of the anatomical points.
2. Cephalograms acquired should have calibration ruler for determination of magnification.

### Exclusion criteria

1. Cephalograms showing excess soft tissue that could interfere with locating anatomical points.
2. Cephalograms of patients with positional errors as reflected by ear rod markers.
3. Cephalograms in which landmarks could not be identified because of motion, resolution disparity or lack of contrast.

Manual tracing of pre-treatment lateral cephalograms of patients was carried out and cephalometric measurements of the 12 parameters were recorded. Similarly, cephalometric measurements of digital images of same cephalogram obtained by the “WebCeph”™ were recorded. All the cephalometric analyses were done by a experienced orthodontist (RKM) with more than 10 years of experience. A maximum of 5 cephalograms were traced per day to avoid the errors due to fatigue.

### Manual tracing

**Fig. 1:**
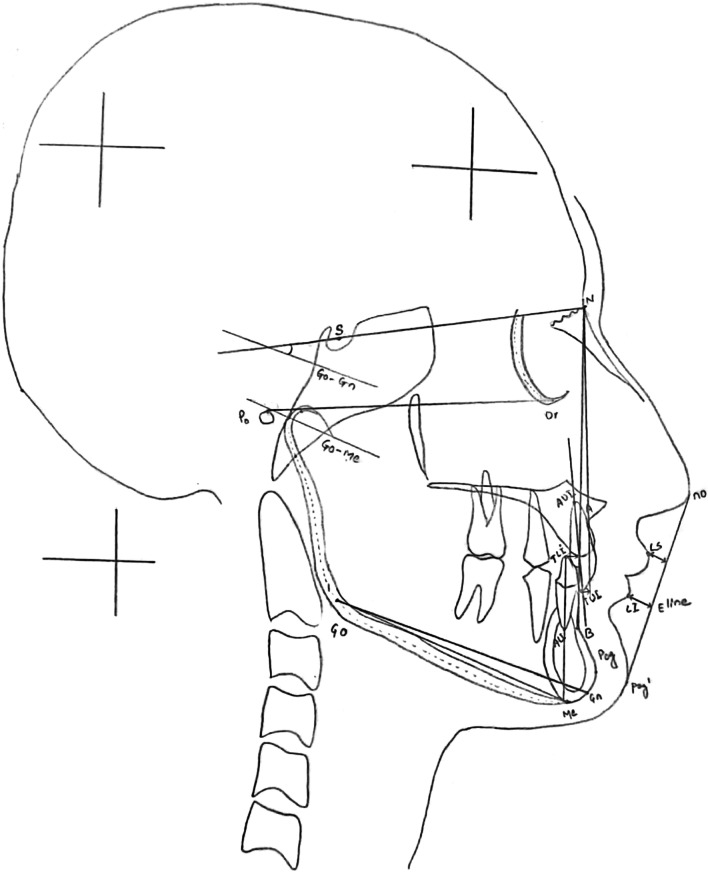
Landmarks [S: Sella, N: Nasion, Po: Porion, Or: orbitale, Go: Gonion, A: Point A, B: Point B, Pog: Pogonion, Me: Menton, Gn: Gnathion, TUI: Tip of Upper incisor, AUI: Apex of Upper incisor, TLI: Tip of lower incisor, ALI: Apex of Lower incisor, no: Tip of nose, Ls: Labrale Superius, Li: Labrale inferius, Pog′: Soft tissue pogonion,] and cephalometric parameters [ SNA, SNB, ANB, S-N to Gn, FMA, U1 to NA (°), U1 to NA (mm), L1 to NB (°), L1 to NB (mm), IMPA/L1 to MP (°), UL to E-line, LL to E-line] used in the study

For manual tracing, hard copies of digital images of same lateral cephalograms will be obtained on 8″ × 10″ radiographic film. Manual tracings were carried on a view box using transilluminated light in a dark room. Each cephalogram was firmly secured to the surface of view box. A sheet of fine grade 0.003″ × 8″ × 10″ matte acetate tracing paper was taped over the X-ray film. Any stray light radiations were eliminated by covering margins of the view box around the radiograph with a black paper. Three orientation marks were placed over the film and these were transferred to the tracing paper for reference. After placing registration points, using a 3H pencil, hard and soft tissue landmarks were traced manually in a predetermined order on the tracing sheet. For bilateral structures and double images, the mid-point was constructed to make a single landmark. A total of 18 anatomical landmarks were plotted on each cephalogram. Measurements of 12 commonly used cephalometric parameters (8 angular and 4 linear) were taken with the help of a millimetre ruler and protractor to the nearest 0.5 mm and 0.5^◦^ respectively (Fig. 1) and (Table 1). Calibration of the actual size of each image in millimetres was done based on measurement of the known distance (10 mm) between the two fixed points of the ruler on the cephalogram. Magnification of the images, if present was calculated. After adding magnification factor to the obtained linear measurements, final values were recorded. All measurements were entered into Microsoft Office Excel 2007 (Microsoft Corp, Redmond, Washington, USA) spreadsheet.

**Table 1:** Description of cephalometric parameters used in the study

Parameters	Description
*Skeletal parameters*	
SNA	Angle formed between points S, N and A
SNB	Angle formed between points S, N and B
ANB	Angle formed between points A,N and B
S-N to Go-Gn	Angle formed between S-N plane and Go-Gn Plane
FMA (Go-Me X Po-Or)	Angle formed between mandibular plane (Go-Me) and Frankfort Horizontal plane (Po-Or)
*Dental parameters*	
U1 to NA (°)	Angle formed between long axis of upper incisor to NA plane
U1 to NA (mm)	Distance between cusp tip of upper incisor to NA plane
L1 to NB (°)	Angle formed between long axis of lower incisor to NB plane
L1 to NB (mm)	Distance between cusp tip of upper incisor to NB plane
IMPA/L1 to MP (°)	Angle formed between long axis of lower incisor to mandibular plane (Go-Me)
*Soft tissue parameters*	
UL to E-line	Distance between most anterior part of upper lip (Ls) to E-line (line connecting no and Pog′)
LL to E-line	Distance between most anterior part of lower lip (Li) to E-line (line connecting no and Pog′)

### Web-based fully automated tracing

**Fig. 2:**
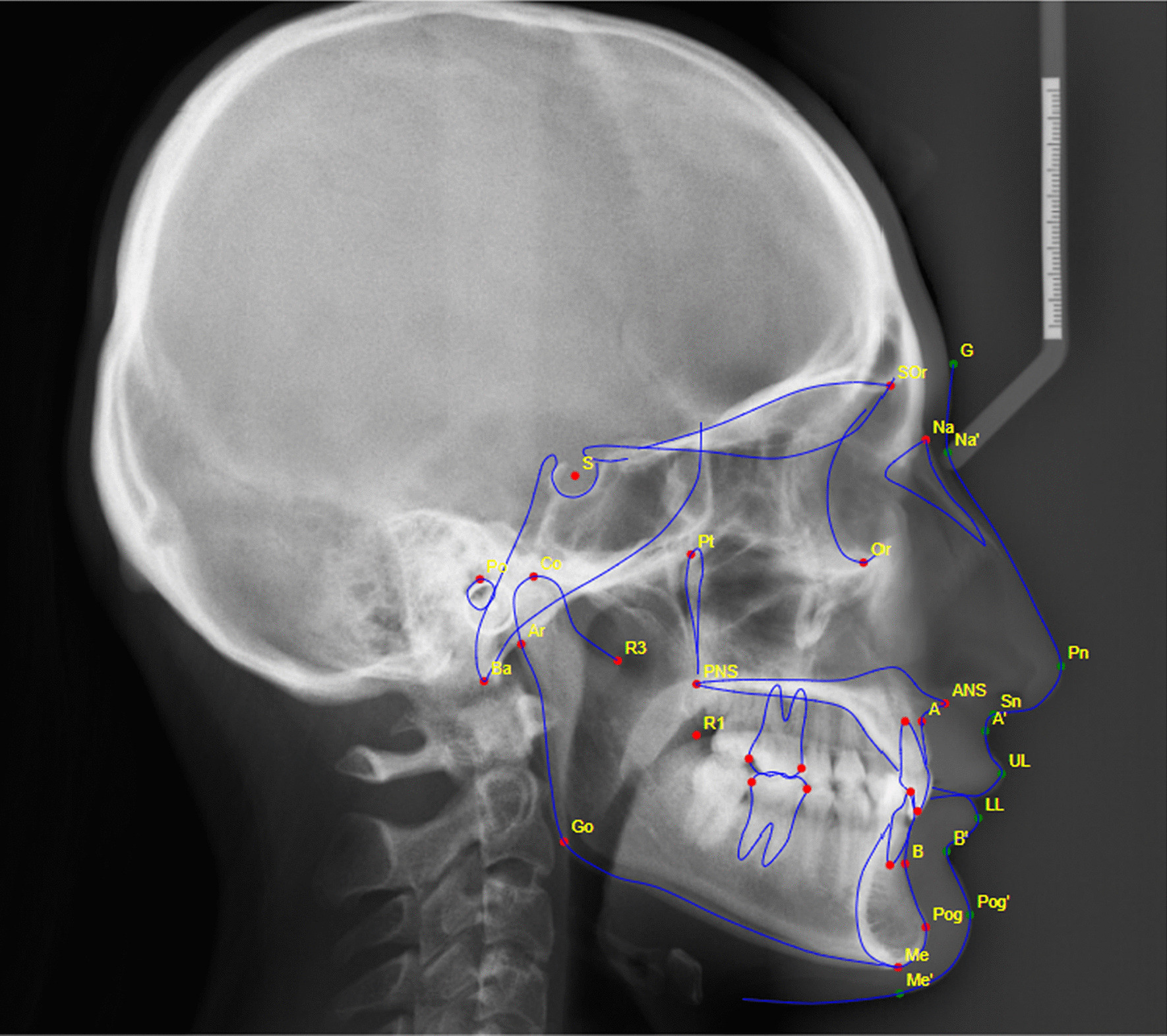
Landmarks and tracing done by AI driven fully automated software “WebCeph”™

For web-based fully automated tracing, an online account was created on the WebCeph™ (https://webceph.com, AssembleCircle Corp., Gyeonggi-do, Republic of Korea) using Google Chrome (Google LLC, California, USA) as standard internet browser. Using the created account, patient profiles were created in the system and digital images of cephalogram were uploaded to respective profiles. After that, using the AI Digitization feature of the WebCeph™ automated landmark identification and tracing by the software was done (Fig. 2). Image was calibrated using the ruler of 10 mm displayed on screen which has to be fitted to the calibration ruler present on the digital image of cephalogram. Finally, the cephalometric measurements value obtained for the different parameters were downloaded in portable document format (pdf) and entered into same Microsoft Office Excel spreadsheet used for entering manual tracing values. Same process was applied for all the 30 digital cephalograms.

To evaluate intra-observer reliability and reproducibility for manual and web-based fully automated AI driven method, 10 radiographs were randomly selected. These cephalograms were retraced manually and cephalometric measurements were obtained with a 10 day interval between evaluations. Similarly, cephalometric measurements of digital images of same cephalogram obtained by the “WebCeph”™ were recorded.

### Statistical analysis

Statistical analysis was carried out using Statistical Package for Social Sciences (SPSS) (version 21.0; IBM, Armonk, NY, USA). Intraclass correlation coefficient (ICC) was used to determine agreement between the measurements obtained from manual tracing and web-based fully automated Artificial Intelligence (AI) driven platform “WebCeph”™ as well as to evaluate intra-observer reliability and reproducibility. ICC value < 0.75 was considered as poor to moderate agreement while an ICC value between 0.75 and 0.90 was considered as good agreement. Agreement was rated as excellent when ICC value > 0.90 was obtained [15].

## Results

ICC values for repeated cephalometric measurements were > 0.9 indicative of very high intra-observer reliability. The ICC values of cephalometric measurements for 12 parameters between manual tracing and web-based fully automated AI driven platform “WebCeph”™ are reported in Table 2. All the measurements had ICC value above 0.75. A higher ICC value > 0.9 was obtained for seven parameters i.e. ANB, FMA, IMPA/L1 to MP (°), LL to E-line, L1 to NB (mm), L1 to NB (°), S-N to Go-Gn whereas five parameters i.e. UL to E-line, U1 to NA (mm), SNA, SNB, U1 to NA (°) showed ICC value between 0.75 and 0.90.

**Table 2:** Intraclass correlation coefficient and 95% confidence interval of cephalometric measurements between manual tracing and web-based fully automated Artificial Intelligence (AI) driven platform “WebCeph”™-skeletal, dental and soft tissue parameters

Parameters	Manual versus WebCeph™	
	ICC	95% CI
SNA	0.879	0.745–0.943
SNB	0.899	0.770–0.954
ANB	0.908	0.747–0.961
S-N to Go-Gn	0.966	0.929–0.984
FMA (Go-Me X Po-Or)	0.914	0.588–0.970
U1 to NA (°)	0.795	0.571–0.902
U1 to NA (mm)	0.850	0.424–0.945
L1 to NB (°)	0.924	0.840–0.964
L1 to NB (mm)	0.919	0.830–0.961
IMPA/L1 to MP (°)	0.915	0.823–0.959
UL to E-line	0.810	0.606–0.909
LL to E-line	0.916	0.826–0.960

ICC, intraclass correlation coefficient; CI, confidence interval

## Discussion

Artificial Intelligence (AI) is one of the milestone achievements of modern-day science which has shown multitude of applications in different fields and orthodontics is no exception in this regard. In early years, the application of AI was limited to clinical diagnosis and treatment [16, 17]. With time, application of AI for automatic identification of cephalometric landmarks was started [18, 19].

As a result, various commercially available fully automated AI driven cephalometric analysis platforms like CephX®, CEFBOT and WebCeph™ have been developed. The major advantage of using these softwares is that multiple cephalometric analyses can be accomplished within seconds after digital cephalogram is uploaded. This significantly improves the efficiency of orthodontists in carrying out cephalometric analysis in routine clinical practice and research.

Errors due to faulty identification of landmarks can result in inaccurate cephalometric interpretation which might lead to errors during orthodontic diagnosis and treatment planning. Hence, it is imperative to assess the accuracy of these fully automated AI driven softwares.

The sample size of this study was selected based on previous studies by H. Alqahtani [11] and TP Silva et al. [13]. Direct-digital images were uploaded for automatic landmark identification by “WebCeph”™ as the use of direct-digital image improved the accuracy of automatic landmark detection as compared to scanned analog image [20]. For assessing the accuracy of “WebCeph”™, cephalometric measurements were used instead of landmark identification in this study because measurements are the end products of cephalometric tracing process and provide data for treatment plan. Also, errors in landmark position used in combination to obtain the measurements might cancel each other out or increase the discrepancy [21, 22].

The parameters (skeletal, dental, and soft tissue) chosen in this study included all the areas of the cephalogram for a more meaningful and reliable comparison. These were commonly used cephalometric parameters needed for orthodontic diagnosis, treatment planning, and evaluation of treatment results (Table 1).

Operator experience is a major factor that can lead to errors during landmark identification. Generally, interexaminer error is greater than intraexaminer error. Hence, all the landmark identification and measurement were carried out by single experienced orthodontist to minimize the error [23].

Various statistical analyses like Pearson correlation coefficient, paired t-test, Band-Altman plot and ICC are commonly used for checking the reliability between different measurements [24]. Zaki et al. [25] conducted a systematic review of statistical methods used to test reliability of medical instruments and concluded that ICC is the most popular method used to assess the reliability of medical instruments measuring continuous outcomes. Hence, we used ICC to determine agreement between the measurements obtained from manual tracing and “WebCeph”™.

**Fig. 3:**
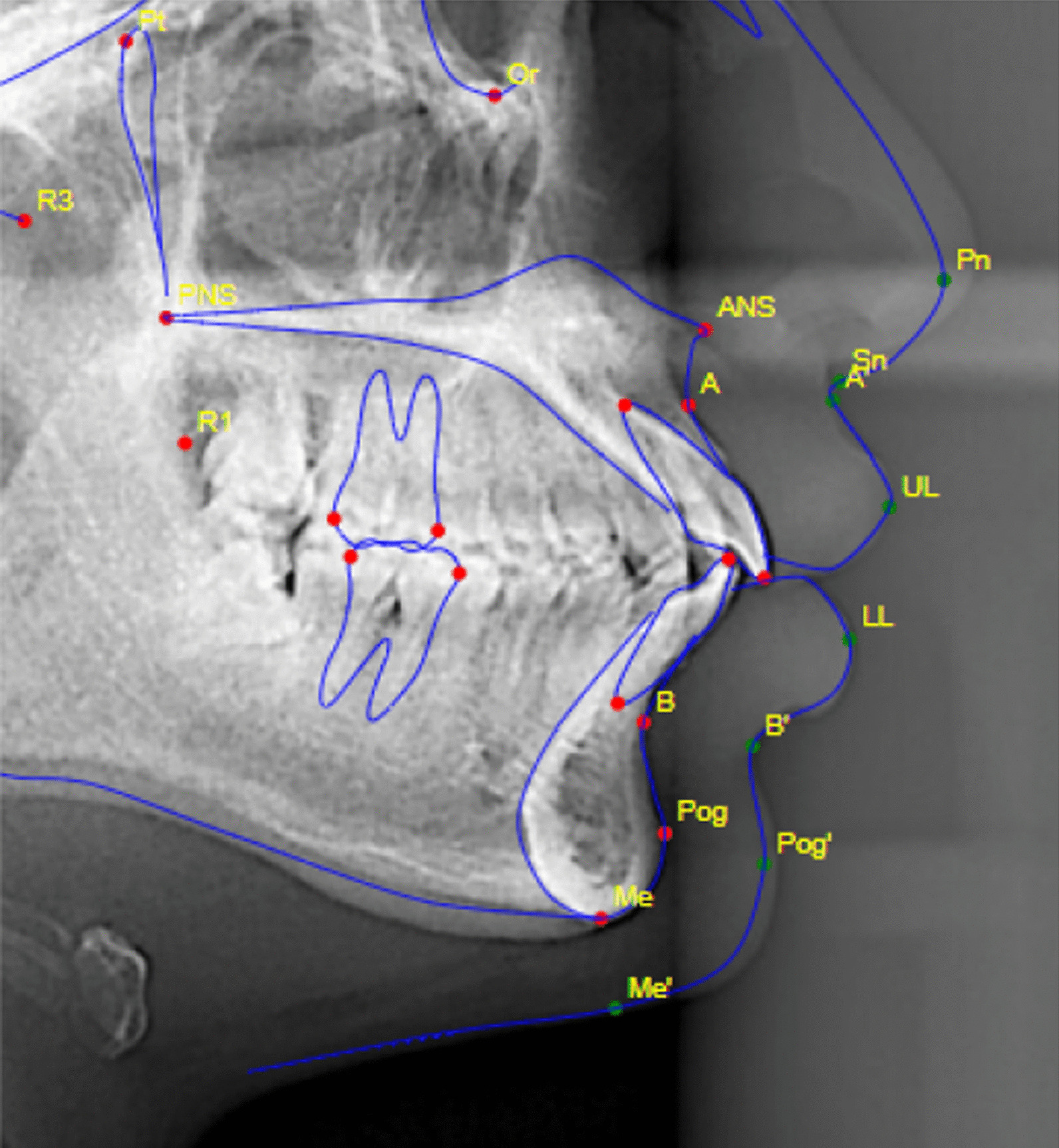
Faulty identification of some landmarks by “WebCeph”™

In our study, all the parameters had ICC value above 0.75. A higher ICC value > 0.9 was obtained for seven parameters i.e. ANB, FMA, IMPA/L1 to MP (°), LL to E-line, L1 to NB (mm), L1 to NB (°), S-N to Go-Gn. However, five parameters i.e. UL to E-line, U1 to NA (mm), SNA, SNB, U1 to NA (°) showed ICC value between 0.75 and 0.90. The reason for lower ICC value may be attributed to faulty identification of landmarks by the software in few cases (Fig. 3).

Recently, few studies have evaluated the accuracy of other AI driven fully automated cephalometric softwares. Alqahtani [12] assessed the reproducibility of 8 linear and 8 angular measurements of cephalogram tracings made with a web-based platform CephX® and tracings made using the FACAD® computer software. He concluded that the measurements obtained by both FACAD® and CephX® softwares are reproducible. Although significant differences were detected for some measurements like SNA, FMA and Pg to NB but all differences were not clinically significant.

Similarly, Meriç and Naoumova [13] compared 12 cephalometric measurements obtained from Dolphin Imaging 13.01 (Dolphin Imaging and Management Solutions, Chatsworth, California, USA), app-aided tracing using the CephNinja 3.51 app (Cyncronus LLC, Washington, USA), web-based fully automated tracing with CephX (ORCA Dental AI, Las Vegas, Nevada, USA) and manual tracing. They found that statistically significant differences were found for cephalometric parameters like GoGn- SN (°), I-NA (°), I-NA (mm), I-NB (°), I-NA (mm) and concluded that fully automatic analysis with CephX needs to be more reliable. However, CephX analysis with manual correction is promising for use in clinical practice because it is comparable to CephNinja and Dolphin, and the analyzing time is significantly shorter.

While, Silva et al. [14] compared 10 cephalometric measurements of Arnett’s analysis obtained from manual tracing method and CEFBOT; an artificial intelligence (AI) based cephalometric software. They found that measurements obtained from two methods were not statistically different.

Orthodontic diagnosis and treatment planning based on the cephalometric measurements obtained from “WebCeph”™ using AI driven fully automated feature can be misleading at times. It is imperative that the landmarks and tracings obtained through fully automated software be supervised by an experienced orthodontist. Alternative option of manual landmark correction provided by “WebCeph”™ may improve the accuracy of cephalometric measurements.

### Limitations and future suggestions

Although adequate number of subjects and parameters were taken in this study, future studies encompassing a greater number of subjects and cephalometric parameter are suggested. Increasing the number of subjects and parameters can assess the reliability of the software with greater accuracy. We found that automated “WebCeph”™ tracing was faster as compared to manual tracing. However, we did not compare the time required for performing cephalometric measurement by two methods and can be analyzed in future studies.

## Conclusion

Based on the findings of this study, it can be concluded that automated cephalometric measurements obtained from “WebCeph”™ are fairly accurate as compared to manual tracing. Apart from quick cephalometric analyses and interpretation, features like cloud based storage of patient’s records, visual treatment simulation and superimposition can make “WebCeph”™ an efficient and promising tool for routine clinical orthodontic practice.

## Data Availability

The datasets generated and analyzed during the current study are not publicly available due to privacy reasons (records of patients), but are available from the corresponding author on reasonable request.

## Abbreviations

AI: Artificial Intelligence
ICC: Intraclass correlation coefficient
SPSS: Statistical Package for Social Sciences
